# Preparation of Porous Stainless Steel Hollow-Fibers through Multi-Modal Particle Size Sintering towards Pore Engineering

**DOI:** 10.3390/membranes7030040

**Published:** 2017-08-04

**Authors:** Francois-Marie Allioux, Oana David, Miren Etxeberria Benavides, Lingxue Kong, David Alfredo Pacheco Tanaka, Ludovic F. Dumée

**Affiliations:** 1 Deakin University, Institute for Frontier Materials, Geelong, VIC 3216, Australia; lingxue.kong@deakin.edu.au (L.K.); ludovic.dumee@deakin.edu.au (L.F.D.); 2 Tecnalia, Energy and Environment Division, Mikeletegi Pasealekua 2, 20009 San Sebastian-Donostia, Spain; oana.david@tecnalia.com (O.D.); miren.etxeberria@tecnalia.com (M.E.B.); alfredo.pacheco@tecnalia.com (D.A.P.T.)

**Keywords:** porous stainless steel hollow-fiber, metal membrane, multi-modal distributions, coalescence, neck formation

## Abstract

The sintering of metal powders is an efficient and versatile technique to fabricate porous metal elements such as filters, diffusers, and membranes. Neck formation between particles is, however, critical to tune the porosity and optimize mass transfer in order to minimize the densification process. In this work, macro-porous stainless steel (SS) hollow-fibers (HFs) were fabricated by the extrusion and sintering of a dope comprised, for the first time, of a bimodal mixture of SS powders. The SS particles of different sizes and shapes were mixed to increase the neck formation between the particles and control the densification process of the structure during sintering. The sintered HFs from particles of two different sizes were shown to be more mechanically stable at lower sintering temperature due to the increased neck area of the small particles sintered to the large ones. In addition, the sintered HFs made from particles of 10 and 44 μm showed a smaller average pore size (<1 μm) as compared to the micron-size pores of sintered HFs made from particles of 10 μm only and those of 10 and 20 μm. The novel HFs could be used in a range of applications, from filtration modules to electrochemical membrane reactors.

## 1. Introduction

Membrane separation processes are well-established alternatives to conventional separation processes and physico-chemical treatments, offering lower energy consumption, compact, and scalable module systems [1]. The unique ability of membrane processes to treat chemically and heat sensitive effluents is of particular importance in the food, pharmaceutical, and biofuel industries [2,3]. Porous membrane elements are designed into a variety of shapes and geometries [4]. Among them, hollow-fiber (HF) membranes, which are self-supported structures, offer specific advantages, such as a higher surface to volume ratio and a uniform flow dynamic across the length of the HFs [2,5,6].

The first porous HFs were made of polymer materials, and were developed almost 60 years ago [7]. Since then, and with the use of novel polymeric materials, polymeric HF membranes have found extensive use in industrial gas separation and water treatment, and in the biotechnology field as well as in the medical sector for blood filtration and treatment . Polymeric HF membranes are produced at low cost and in large quantities via a dry–wet spinning process, and can be assembled as one module, which can be composed of thousands of polymeric HFs [3,8]. However, polymeric-based membranes typically exhibit lower tolerance to suspended abrasive particles, chemical cleaning, and steam sterilization procedures as compared to inorganic-based membranes [9]. The development of membranes with a high tolerance to cleaning, disinfection, and sterilization procedures could therefore drastically increase the reliability and life expectancy of such separation systems [3,10].

In a context where processes must achieve high durability and reusability, inorganic-based membranes offer unique advantages, such as high mechanical and thermal resistance, as well as increased stability in harsh chemical and abrasive environments [11,12]. In particular, metal porous HFs exhibit higher mechanical strength and ductility as opposed to the more brittle ceramic and carbon porous HFs [13]. A number of metal and alloy-based materials are also particularly resistant to oxidative chemical and thermal cleaning procedures, providing outstanding reusability characteristics [12,14]. To date, most of the work on the development of metal HFs has been focused on corrosion-resistant metals, such as stainless steel (SS) or titanium alloys, for separation applications [5,15,16]. However, porous HFs made of highly electrically conductive and electro-catalytic materials, such as nickel and copper (outer diameter between 250 and 700 μm), were also prepared and used in electro-membrane reactors for the electrochemical reduction of carbon dioxide or for methane reforming process [8,17,18]. In addition, porous HFs can be used as supports for selective thin films such as palladium–silver (Pd–Ag) materials for water gas shift reactions [19]. Inorganic metal HF membranes (outer diameter ≤ 3 mm) were successfully prepared using different fabrication processes, among which the dry-wet spinning process is commonly preferred [20,21]. The fabrication of inorganic HF membranes is a process with multiple steps whereby a high viscosity dope containing up to 80 wt % of suspended inorganic particles is spun [5,18,20]. Then, the extruded, or so-called green HFs, undergo a series of thermal treatments to remove the polymer binder and simultaneously start the sintering process between metal particles. The key properties of HF membranes and filters are their porosity and mechanical strength, which are directly related to the number, relative density, and volume of necks generated between the particles [22,23]. The stages of the sintering process include: (i) initial neck formation, (ii) growth at the contact point between the particles, and (iii) densification of the structure and ultimately full coalescence of the particles . While the enlargement of the neck area promotes the mechanical strength of the material, the open porosity is also reduced, limiting the materials’ performance in separation [24]. One strategy to address this issue is therefore to increase the initial number of contact points per particle in order to form a higher density of small necks during the initial sintering stage. The density of the necks can typically be enhanced by using multi-modal particle distributions in order to increase the packing density of the green material, which is highly dependent on the shape and size distribution of the particles [25]. However, the sintering behaviour and kinetics of the mixed powder matrix will differ from conventional sintering models, where mono-sized spherical particles are normally used. New model developments are therefore required in this area of research [25].

In this study, porous SS HF membranes were prepared by the dry-wet spinning process. The green HFs were composed of a mixture of two powders of different sizes in order to control the densification rate during the sintering process and develop ordered porous structures. The impact of the spinning dope composition and sintering conditions on the final HF morphologies, pore sizes, and mechanical and electrical properties were systematically studied. Pure water permeation experiments were also performed in a dead-end filtration module, and the results were compared to sintered SS HFs obtained from single-sized particles. This work opens the door to the development of new types of inorganic hierarchical porous HF structures with enhanced properties for separation applications, where a better compromise between porosity and mechanical strength is achieved.

## 2. Results and Discussion

### 2.1. Metals Powders and Polymer Binder Characterisation

The studies of the shape, roughness, and density of the powders are important steps, since such characteristics will influence the mechanical properties and porosity of the final sintered materials [26].

**Figure 1:**
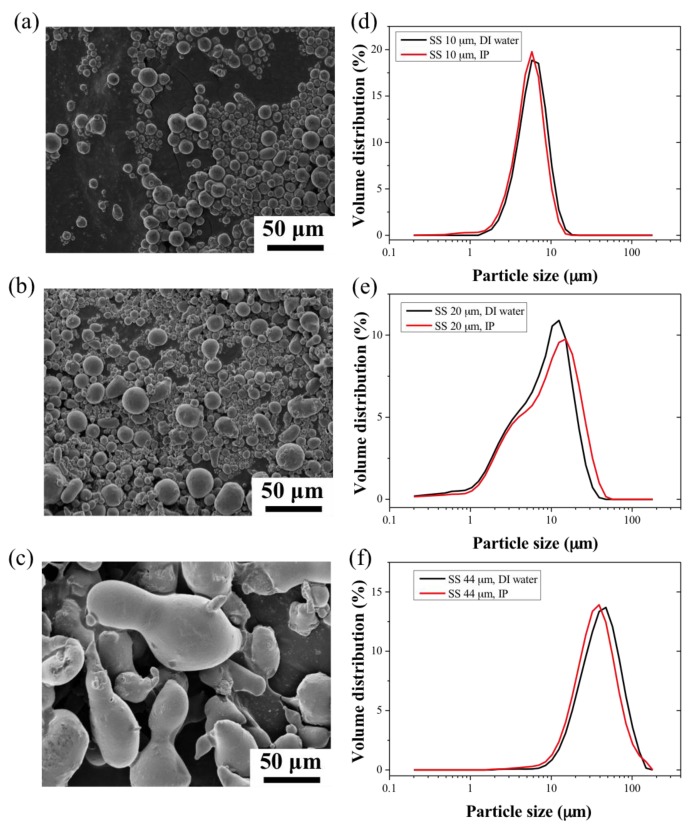
SEM images of (**a**): the 10 μm stainless steel (SS) powder, (**b**): the 20 μm SS powder and (**c**): the 44 μm SS powder. (**d**–**f**) are their respective size measurements in distilled (DI) water and isopropanol (IP).

**Figure 2:**
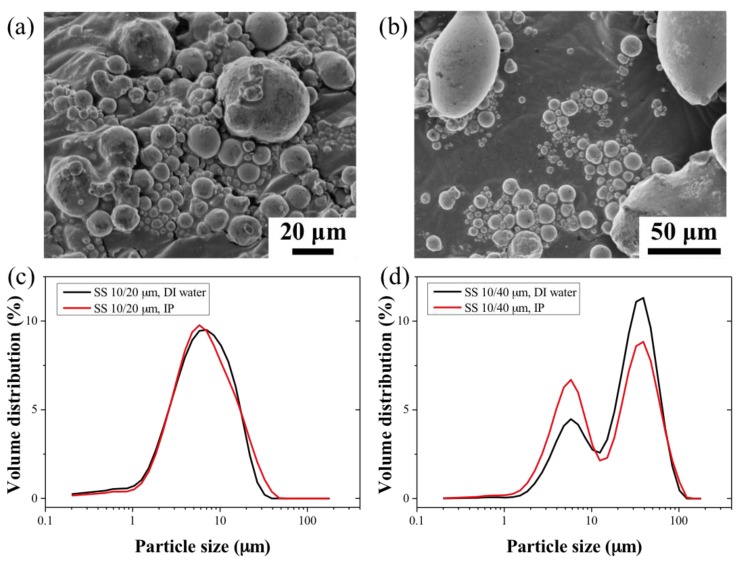
SEM images of (**a**): the 10/20 μm SS powder blend and (**b**): the 10/44 μm SS powder blend. (**c**,**d**) are the respective size measurements of the powder blends in DI water and isopropanol (IP).

The 10 and 20 μm 316L stainless steel particles, SS_10_ and SS_20_ respectively, exhibited smooth surfaces with spheroidal shapes and an aspect ratio of 1.09 ± 0.05 and 1.13 ± 0.1, respectively. On the other hand, the shape distribution of the 44 μm 316L stainless steel particles (SS_44_) was irregular (aspect ratio of 2.17 ± 0.3) and showed greater surface roughness. The particle size distribution of the SS powders and blends was measured in distilled (DI) water and isopropanol (IP) to evaluate potential aggregation mechanisms in the different dispersants. The distributions in both solvents were similar; the SS_10_ powders showed a narrower size distribution as compared to the SS_20_ and SS_44_ particles, which were 40% and 20% wider, respectively. The use of water resulted in little or no clumping of the particles as seen in Figure 1d–f. The information on particle size distributions is shown in Table S1 for the calculated equivalent spherical diameters. The particle size distributions of the SS_44_ particles were found to be almost twice larger than the specifications of the manufacturer, with 90% of the distribution in volume of the particles (d0.9) lying below 72 ± 1.1 μm in water. The surface area of the powder samples was measured using Brunauer-Emmett-Teller (BET) analysis, the data are reported in Table S2. The BET surface areas of the SS_10_ and SS_20_ particles were similar; 0.034 and 0.036 m^2^.g^−1^, respectively. However, the BET surface area of the SS_44_ particles was found to be greater, 0.052 m^2^.g^−1^, which might be due to the textured surface of the SS_44_ particles. The weight ratio of the SS particle blends can therefore be expressed in terms of surface area ratio, corresponding to 72:28 (%). and 92:8 (%) for the SS_10_:SS_20_ and SS_10_:SS_44_ blends, respectively. Furthermore, the particle size distributions of the SS powder blends (Figure 2c,d) indicated that the particles did not aggregate in the solutions. The SS_10_ and SS_44_ powders exhibited similar average absolute and relative densities. However, the SS_20_ powders showed a higher relative density due to the wider particle size distribution (Table S2).

**Figure 3:**
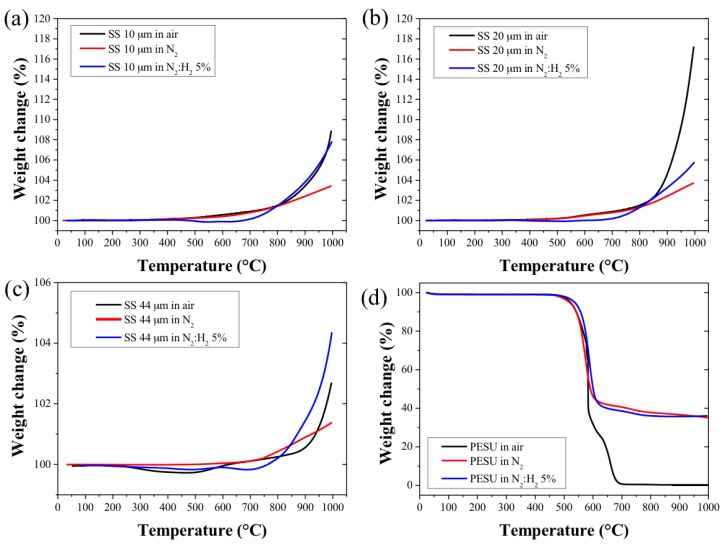
Thermogravimetry (TGA) thermal profiles measured in air, inert, and reducing atmosphere of (**a**): 10 μm SS powder, (**b**): 20 μm SS powder, (**c**): 44 μm SS powder and (**d**): PESU polymer binder.

During the sintering process, the bonding of the metal particles occurred through the formation of a sintered neck area between the particles. However, sufficient neck formation required the reduction of the surface oxides during the early stages of the sintering [27]. In order to study the oxidation behavior of the SS particles at high temperature, thermogravimetry (TGA) experiments were performed, and consisted of heating a small bed of metal particles (10 mg) to 1000 °C with a heating rate of 10 °C min^−1^ in air, N_2_, and N_2_:H_2_ (95:5 *v/v* %) (Figure 3a–c). These gases were chosen in order to mimic the environment of the furnace during the sintering of the green HFs. In particular, SS alloys become susceptible to corrosion and oxidation when exposed to a high temperature, typically referred to as the sensitization temperature [28,29]. In air and N_2_ atmospheres, the sensitization temperatures of the SS_10_ and SS_20_ were found to be 310 °C and 500 °C, respectively, and as high as 650 °C for the SS_44_. The TGA curves of the SS_44_ in air revealed the presence of residual organic contaminants, probably resulting from the fabrication process and associated with the slight weight loss from 200 to 400 °C (Figure 3c).

The weight gains of the three powders were found to occur at higher temperatures in reducing N_2_:H_2_ atmosphere with sensitization temperatures above 650 °C for the SS_10_ and SS_20_ particles and above 750 °C for the SS_44_ particles. The finest SS powders (SS_10_ and SS_20_) showed the greatest weight gain upon reaching the 1000 °C mark due to the higher surface area to volume ratio of the particles in comparison to the larger SS_44_ particles. The SS_20_ and SS_44_ powders were found to be more prone to oxidation as compared to the SS_10_ powder due to their as-received oxidized surface state, which was analysed by EDS analysis. The oxygen content was 1.7 and 1.5 ± 0.1 wt % for the SS_20_ and SS_44_ powders, respectively, and was below the detection limit for the SS_10_ powder. Similarly, the carbon content of the SS_20_ and SS_44_ powders was found to be higher; 7.4 and 8.5 ± 0.3 wt %, respectively. Furthermore, the high carbon content of the SS_44_ particles could also explain the lower weight gain at high temperature. The weight gain was associated with the formation and growth of stable surface metal oxide compounds across their surface [30,31]. The weight gain of the particles observed in N_2_ and N_2_:H_2_ atmospheres was due to the uptake of nitrogen atoms by the SS materials, leading to the formation of nitride species [32,33,34]. The kinetics of oxide formation and growth were shown to be greatly increased in air; however, the presence of oxygen did not seem to influence the sensitization temperature of the different particles.

The thermal characterization of the polymer binder is also of particular importance for the fabrication of metal HFs, since the polymer burn-out should occur below the sensitization temperatures of the metal particles in order to avoid any reaction and oxidation with the metal particles. In addition, the polymer matrix should be maintained at a lower temperature to ensure the structural integrity of the HF during the polymer de-binding step. Figure 3d shows the thermal decomposition of PESU in air, inert (N_2_), and reducing (N_2_:H_2_ 5%) atmosphere. The decomposition of PESU occurred abruptly at 600 °C below the temperature corresponding to the sensitization temperature of the SS particles. The TGA profile of the green SS_10_ HF in air atmosphere is presented in Figure S1, and shows a similar decomposition of the PESU matrix during the de-binding step at 600 °C. However, a weight gain was observed during the thermal treatment, suggesting that sensitization could not be avoided during the de-binding step. The thermal treatment of the green HF was therefore performed in reducing atmosphere in order to prevent the oxidation of the particles, promote metal-to-metal contact, and form neck area between the metal particles [27,31]. However, the thermal decomposition of PESU in inert and reducing atmosphere (Figure 3d) revealed that up to 35 wt % of organic residues remained. The sintered HFs were therefore thoroughly cleaned first with ethanol and distilled water before any characterization tests.

### 2.2. Green and Sintered HF Morphologies

**Figure 4:**
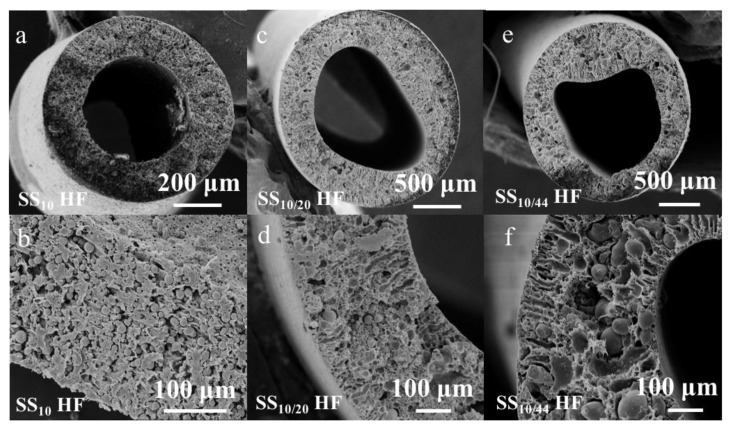
Cross-sectional SEM images of (**a**,**b**): the SS_10_ green hollow-fibers (HFs); (**c**,**d**): the SS_10/20_ HFs; (**e**,**f**): the SS_10/44_ HFs.

The dopes comprised of SS particles and PESU polymer binder were then extruded to form the green HF material. The SEM images of the green HFs are shown in Figure 4. The average outer diameter of the green HFs made of SS_10_ particles was found to be 1.23 ± 0.03 mm, and to exhibit circular cross-sectional morphology with a uniform distribution of SS_10_ particles within the PESU matrix. The outer diameters of the green SS_10/20_ and SS_10/44_ HFs, 2.24 ± 0.05 and 2.17 ± 0.45 mm respectively, were found to be larger than the SS_10_ HFs due to a shorter air gap, resulting in less stretch and elongation stress on the forming HFs [3]. The ellipsoidal shape and irregular circularity of the green SS_10/20_ and SS_10/44_ HFs were attributed to aggregates and clusters of metal particles of different sizes, which may have formed in the dope during the degas step prior to spinning. The circularity of the green HFs could be further improved by increasing the bore flow rate in order to break the aggregates in the dope solution. The SEM images reveal the presence of macro-voids and finger-like structures, starting from both sides of the HFs exposed to the bore and external coagulation liquid and going toward the inner core of the HFs. This structure is more pronounced for the SS_10/20_ and SS_10/44_ HFs due to the presence of clusters of metal particles inside the continuous PESU phase.

**Figure 5:**
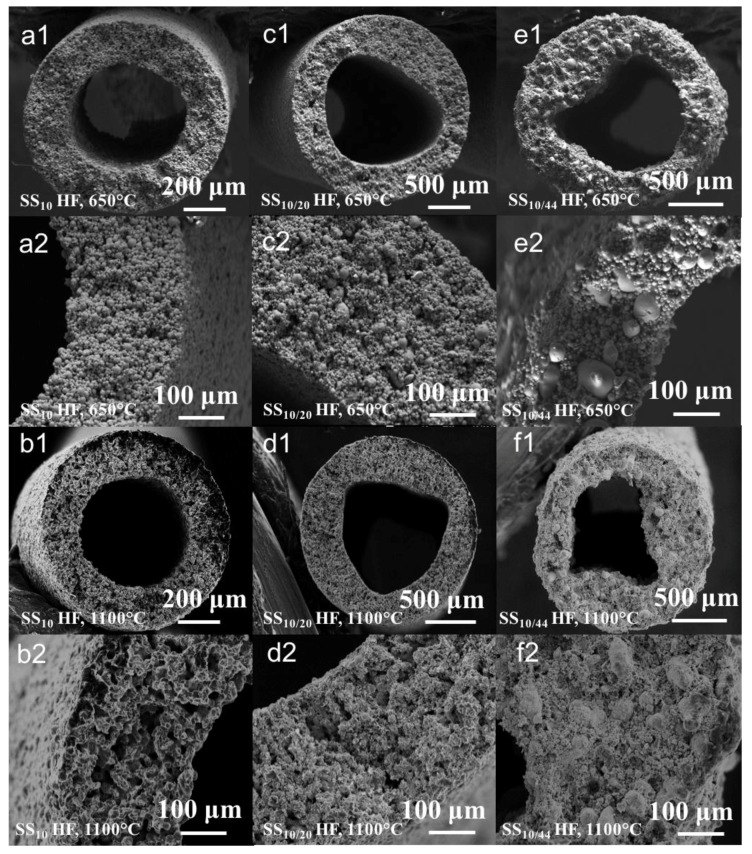
Cross-sectional SEM images of the sintered SS_10_ HFs for 90 min at 650 °C (**a1**,**a2**) and 1100 °C (**b1**,**b2**); sintered SS_10/20_. HFs for 90 min at 650 °C (**c1**,**c2**) and 1100 °C (**d1**,**d2**); sintered SS_10/44_. HFs for 90 min at 650 °C (**e1**,**e2**) and 1100 °C (**f1**,**f2**).

As seen in Figure 5, the sintered HFs retained their original lumen shapes upon thermal treatment. The HFs made of SS_10_ and SS_10/20_ particles exhibited smooth surfaces, whereas the macro-voids present across the wall of the green HFs also remained after the thermal treatment. On the other hand, the HFs made of SS_10/44_ particles presented extremely rough surfaces, with some of the largest particles pointing out of the structure. This geometry may be due to the shear displacement of the largest particles across the matrix of small particles during the sintering [35]. As opposed to the SS_10_ and SS_10/20_ HFs, the cross-sectional SEM images of the SS_10/44_ HFs revealed a denser structure. The original macro-voids observed in the green HFs were also absent after sintering, as expected due to the high diffusion rate of the smaller particles, which were able to fill the macro-voids during the thermal treatment. The shape of the metal particles present in the SS_10_ and SS_10/20_ green HFs became indistinguishable when sintered at 1100 °C, indicating that the sintering process was completed, which was not the case for the SS_10/44_ HFs where the shapes of the particles were still well-defined (Figure 5(f1,f2)). The SEM images of the sintered HFs across the whole range of sintering temperature and time are displayed in Figures S2–S4.

### 2.3. Sintered HF Properties

**Figure 6:**
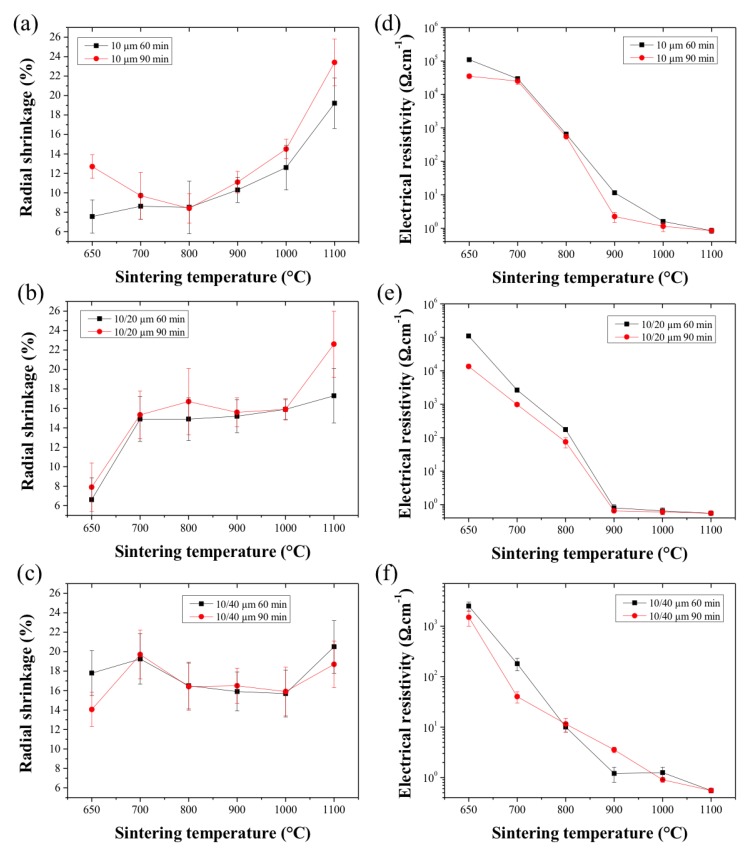
Radial shrinkage from green to sintered HFs as a function of the sintering time and temperature. (**a**): SS_10_ HFs, (**b**): SS_10/20_ HFs and (**c**): SS_10/44_ HFs. (**d**–**f**) are the electrical resistance of the SS HFs as a function of the sintering temperature and time for the SS_10_, SS_10/20_ and SS_10/44_ HFs.

The radial shrinkage was measured based on the SEM cross-sectional images, and the measurement results (%) are shown in Figure 6. The sintered HFs exhibited isotropic radial shrinkage strongly dependent on the sintering temperature. A slight increase in shrinkage values was noticed for the SS_10_ and SS_10/20_ HFs when sintered for a longer duration (90 min) as shown in Figure 6a,b. The radial shrinkage of the SS_10_ HFs remained below 10% up to 900 °C, which corresponded to the first stage of the sintering process only. On the other hand, the SS_10/20_ and SS_10/44_ HFs showed greater radial shrinkage (>15%) across the range of sintering temperature. This effect was particularly noticeable for the SS_10/44_ HFs due to the difference in size and shapes of the metal particles, which promoted the densification of the structures across the whole range of sintering temperature [24].

As observed in cross-sectional SEM images (Figure 5(a1,c1,e1)), the denser structures resulting from the thermal treatment at a lower temperature can be explained by the incomplete degradation of the polymer during the de-binding step. This observation is supported by the EDX chemical analysis and SEM images (Figure S5 and Figure 5), which revealed that the de-binding step was incomplete at the lowest temperatures (650 and 700 °C), and that a large amount of carbon remained in the structure. The densification of the structure at a lower temperature is therefore not sufficient to induce the collective sintering of the metal particles due to the residual carbon hampering particle-to-particle contacts and particle neck formation. This effect may be alleviated by using a different polymer binder with a lower decomposition temperature or by prolonging the duration of the de-binding phase.

The electrical resistance measurements (Figure 6d–f) of the HFs sintered at a temperature lower than 900 °C indicated that the densification of the structure is not sufficient to create a significant conductive pathway across the inter-particle neck area. The enhanced mechanical strength of the SS_10/20_ and SS_10/44_ HFs at lower temperatures can be explained from the increased initial neck growth of the smallest particles to the largest particle. The initial neck growth between the smallest and largest metal particles is able to form a scaffold maintaining the structural integrity of the HFs even at the lowest sintering temperatures. Across all of the HF samples, the electrical resistance was drastically reduced from 900 °C of sintering temperature, indicating that the neck areas were able to form a continuous and conductive scaffold across the HFs [20,21]. No significant differences were, however, found between the HFs sintered for 60 or 90 min, suggesting that a percolation threshold had been reached by that time.

**Figure 7:**
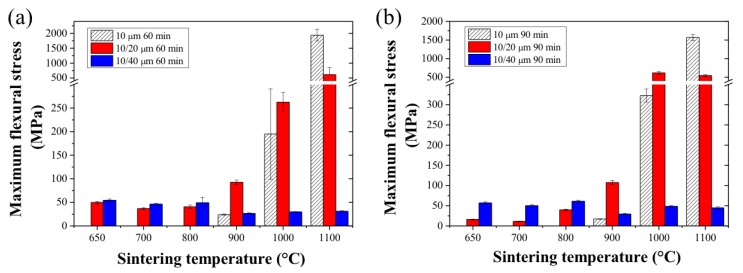
Maximal flexural stress of the SS HFs as a function of the sintering temperature with (**a**): HFs sintered for 60 min and (**b**): HFs sintered for 90 min.

The mechanical properties of the sintered SS HFs are therefore highly related to the dope composition and to the thermal treatment conditions, including the sintering temperature, duration, and atmosphere. The mechanical strength of the SS_10_ HFs was found to be acceptable for handling and testing only when sintered above 900 °C (Figure 7a,b) with a maximal flexural stress of 23.9 ± 1.2 and 17.2 ± 0.9 N mm^−2^ when sintered for 60 and 90 min, respectively. On the other hand, the SS_10/20_ and SS_10/44_ HFs exhibited sufficient mechanical strength right from the lowest sintering temperature (650 °C), with a value above 37 N mm^−2^ when sintered for 60 or 90 min, and values slightly lower for the SS_10/20_ HFs when sintered for 90 min (<40 N mm^−2^). In these conditions, the extended sintering time was therefore unable to make up for the lower sintering temperature and to achieve a significant degree of bonding between the metal particles. The SS_10_ and SS_10/20_ HFs exhibit exceptional mechanical strength and flexibility when sintered above 1000 °C. The maximal flexural stress of the SS_10_ HFs sintered at 1000 °C exceeded 200 and 300 N mm^−2^ for a sintering time of 60 and 90 min, respectively, while the SS_10/20_ HFs showed values twice as high as the SS_10_ HFs. SS_10_ HFs sintered at 1100 °C showed a further mechanical strength improvement, with values increased by up to 5 and 10 times depending on the sintering time (Figure 7a,b). However, the SS_10/20_ HFs sintered at 1100 °C for 60 or 90 min did not show any significant mechanical strength improvement. This lack of improvement indicates that the densification of the structure is at a maximum [24]. The mechanical strength of the SS_10/44_ HFs sintered at the same temperatures were, however, found to be significantly lower (<60 N mm^−2^) compared to the SS_10_ and SS_10/20_ HFs. Although the densification of the SS_10/44_ system occurred at lower temperatures and reached a maximum sooner, the lack of coalescence between the large and the small particles formed a more rigid and denser structure. The difference in shape and size between the two SS powders promoted the densification of the systems, with the small particles filling the pores between the large particles. Therefore, it may be preferential to decrease the size ratio difference between the particles as well as to work with uniform spherical particles. The SS_10_ and SS_10/20_ HFs fabricated in this work exhibited similar mechanical performance when compared to previous studies available in the literature; however, the mechanical properties of the SS_10/44_ were found to be inferior [20,34,36].

### 2.4. Pore Size Distribution and Water Permeance

**Figure 8:**
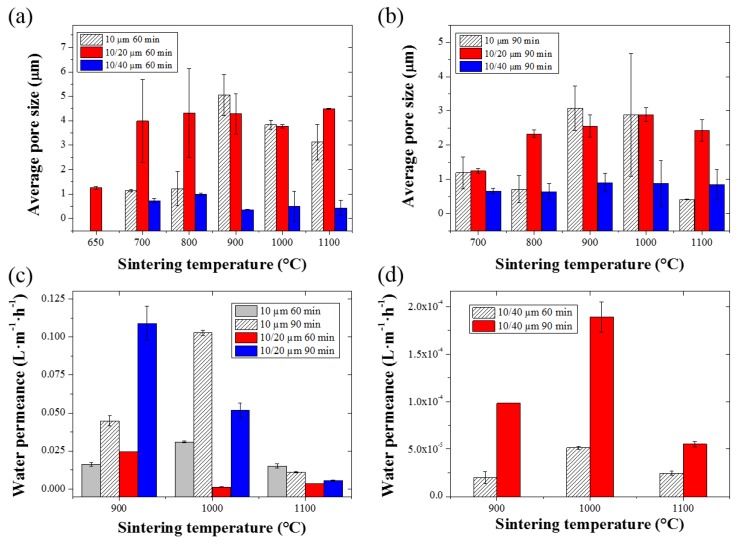
Average pore size measurements of the SS HFs as a function of the sintering temperature, for (**a**): 60 min and (**b**): 90 min sintering time. Water permeance values of (**c**): the SS_10_ and SS_10/20_ HFs as a function of the sintering temperature and time. (**d**): Pure water fluxes of the SS_10/44_ HFs as a function of the sintering temperature and time.

The average pore sizes of the sintered HF membranes, which correspond to the average dimension of the inter-particle domains, were measured by capillary flow porometry. Capillary flow porometry techniques have the advantage to be able to measure the open pores across materials, and is particularly suited to micron-ranged pore distributions [37]. The average pore sizes of the sintered HFs are presented in Figure 8a,b. The SS_10_ and SS_10/20_ HFs sintered at 900 °C presented a similar average pore size—around 5 μm—for a sintering time of 60 min, and around 3 μm when sintered for 90 min. The same trend was observed for a sintering temperature of 1000 °C, with similar average pore sizes between the SS_10_ and SS_10/20_ HFs, and smaller values when sintered for 90 min (Figure 8b). However, the SS_10/20_ HFs sintered at 1100 °C presented larger pore sizes as compared to the SS_10_ HFs. The average pore size of the SS_10_ HFs sintered at 1100 °C for 90 min was reduced to 0.5 μm as opposed to the average pore size of 2.4 ± 0.3 μm found in the SS_10/20_ HFs. The average pore sizes of the SS_10/44_ HFs were found to be below 1 μm for a sintering temperature ranging from 900 to 1100 °C without significant effect on the sintering time. The smaller average pore size of the SS_10/44_ HFs was related to the greater size difference between the two series of particles, which led to a denser structure with smaller pores [24]. A general aspect of sintering is that as the sintering time and temperature increase, the coalescence between the particles is promoted [38]. However, in the case of SS_10/20_ HFs, the maximum density was limited due to the larger particles within the system. As opposed to the SS_10_ HFs, the increase in sintering temperature and time therefore promoted the creation of larger pores in the SS_10/20_ HF structure [38]. On the other hand, the SS_10_ HFs could potentially form dense materials by further intensification of the sintering conditions [18].

Pure water fluxes were measured across a range of pressures varying from 0.2 to 1 bar. The pure water flux measurements exhibited a linear relationship with respect to the applied pressure, and are shown in the supplementary materials (Figure S6). Membrane permeances as a function of the sintering temperature and time are shown in Figure 8c,d. The SS_10/20_ HFs sintered at 900 °C for 90 min exhibited the greatest water permeance among all of the SS HFs (Figure 8c). The SS_10/20_ HFs water permeance values were shown to progressively diminish with respect to the sintering temperature due to the densification process. The highest water permeance, corresponding to the SS_10_ HFs, was attained at a sintering temperature of 1000 °C and for a sintering time of 90 min, likely due to an increased open porosity across the structure. The SS_10_ HFs water permeance drastically diminished at the sintering temperature of 1100 °C, which was therefore considered to be close to the full densification of the material. The SS_10/44_ HFs also presented the lowest water permeance due to their denser structures and smaller pore size distributions, as previously reported (Figure 8d).

## 3. Materials and Methods

### 3.1. Metal Particles and Chemicals

The 10 μm 316L stainless steel (SS_10_) was purchased from Sandvik Osprey Ltd., Neath, UK, while the 20 μm (SS_20_) and 325 mesh size (SS_44_, 44 μm) 316L SS powders were sourced from Huarui Group Ltd., Hangzhou, China. Poly(ethersulfone) (PESU, Ultrason^®^, BASF, Tarragona, Spain) was dried in a vacuum oven at 120 °C for 6 h before being used as the polymer binder. N-Methyl-2-pyrrolidone (NMP, ACS reagent ≥99.0%, Sigma-Aldrich, Madrid, Spain) was chosen as a solvent for PESU and used as received. Distilled water (DI) and isopropanol (IP, ACS reagent ≥99.5%, Sigma-Aldrich, Madrid, Spain) were used during the green HFs drying procedure and for the determination of the metal particle size distributions.

### 3.2. Preparation of the Green HFs

Spinning dopes were prepared by first dissolving the polymer binder into NMP for more than 24 h on a roller tube mixer. Subsequently, the metal particles were added in multiple steps and finally the mixture was stirred overnight. The spinning dope mixture was then loaded into the spinning pump and degassed by applying a vacuum 24 h before the date of the spinning experiment. The compositions of the spinning dopes were as follows: 70 wt % of metal particles, 7.5 wt % of polymer binder, and 22.5 wt % of NMP. In the spinning experiments, where a mixture of SS particles of different size distributions was used, the blend of SS particles comprised 60 wt % of SS_10_ powder and 40 wt % of either SS_20_ or SS_44_ powder.

The spinning setup allowed the spinning of green HFs of various geometries by controlling different process parameters such as air gap (cm) and dope and bore flow rates (mL h^−1^). The temperature was maintained at 25 °C through heating bands wrap around the spinneret assembly and spinning line. The spinning conditions are listed in the Supporting Information section’s Table S3. The spinneret used had an inner and outer diameter of 0.7 and 1.3 mm, respectively.

### 3.3. Drying and Thermal Treatments of Green HFs

The green HFs were firstly kept in a DI bath for 24 h followed by an IP bath for another 24 h in order to exchange the NMP solvent. Finally, the green HFs were dried at room temperature for 24 h. Once dried, thermal treatments were performed in a tubular furnace (model GSL-1100 X, MTI Corporation, Richmond, CA, USA) in order to remove the PESU polymer binder and sinter the SS particles.

The polymer was removed at 600 °C for 60 min followed by a sintering process with temperatures ranging from 650 to 1100 °C for 60 or 90 min at a heating rate of 5 °C min^−1^ under a reducing atmosphere nitrogen/hydrogen mixture (N_2_:H_2_, 85:15 *v/v* %). The gas was first flown 30 min before the thermal treatment in order to purge the air from the furnace, and was maintained at 1 dm^3^·min^−1^ during the thermal treatment.

### 3.4. Materials Characterization

The morphology of the metal particles and the green and sintered HFs was characterized by Scanning Electron Microscopy (SEM, JEOL Neoscope, JCM-5000, Peabody, MA, USA). The elemental distributions on the surface of the samples were evaluated by an Electron Dispersive X Ray Spectroscopy (EDS) analysis with an Oxford detector on a JEOL JSM 7800F model. SEM imaging (surface and cross-section) was performed at 10 keV and a 10 mm working distance, while surface elemental mapping was performed at 20 keV and 10 mm working distance.

The particle size distribution of the metal powders dispersed in water or isopropanol were analysed by Dynamic Light Scattering (DLS) using a Mastersizer 2000 (Malvern Instrument, Worcester, UK). The aspect ratio of the metal particles was determined using ImageJ software (version 1.50i). The specific surface area of the metal powders was measured by the BET method using a TriStar 3000 instrument (Micromeritics, Norcross, GA, USA). The average absolute and relative densities of the metal powders were determined with a 50 mL pycnometer bottle using ethanol as the wetting liquid. The temperature-dependence of the wetting fluid was taken into account. Thermogravimetry (TGA) experiments were performed under air, N_2_, and N_2_:H_2_ mixture (95:5 *v/v* %) using a Q50 TGA (TA instrument, New Castle, DE, USA). During the TGA experiments, the gas flow was maintained at 60 cm^3^·min^−1^, and the heating rate was fixed at 1, 5, or 10 °C·min^−1^.

The pore size and pore size distribution were measured using a capillary flow porometer (Porometer 3GZH Quantachrome Instruments, Boynton Beach, FL, USA) after wetting the sintered HFs with Porofil^®^ (Quantachrome Instruments) wetting solution. The electrical resistivity of the green and sintered HFs was determined using an electrical resistivity cell with a variable resistor. Three-point bending tests were performed on a DMA Q800 (TA instrument, USA) in order to characterize the mechanical resistance of the HFs. The stress rate was set at 0.5 N·min^−1^, and the temperature keep at 25 °C. The bending stress or flexural stress was calculated using the following equation:

(1) $$\sigma =\frac{8FKD_{0}}{\pi \left(D_{0}^{4}-D_{i}^{4}\right)}$$

where σ is the bending stress (MPa), *F* is the measured maximal flexure load (N), *K* is the support span (mm), and $D_{0}$ and $D_{i}$ are the outer and inner diameters of the HFs (mm), respectively.

Pure water permeation tests were performed in a lab-scale polyurethane module. The metal HF membranes with an effective length of 40 mm were first cleaned with ethanol, flushed thoroughly with distilled water, and dried at room temperature. The metal HF membranes were then placed into the module and the distilled feed water was circulated onto the membranes and forced through the membrane walls under pressure varying from 0.2 to 1 bar. The pure water flux of the metal HF membranes was calculated using the equation:

(2) $$J=\frac{Q}{A_{m}}=\frac{Q}{n\pi D_{HF}l_{eff}}$$

where *J* is the membrane pure water flux (L m^−2^·h^−1^), *Q* is water flux rate (L h^−1^), $A_{m}$ is the effective membrane surface area (m^2^), n is the number of HFs, $D_{HF}$ the outer diameter, and $l_{eff}$ is the effective length of the HFs.

## 4. Conclusions

Macro-porous SS HFs were successfully fabricated by the dry-wet spinning of a dope comprised of SS metal powders of different sizes and a polymer binder. A systematic study of the effect of the sintering parameters on the starting materials and sintered HFs was performed. Both the mechanical strength and water permeance of the HFs made of different sized SS particles were improved at a low (900 °C) sintering temperature due to the increased neck density offered by the multi-modal particles’ distribution. At a sintering temperature higher than 1000 °C, SS particle repacking during the thermal treatment gave rise to a denser structure with a lower water permeance. The porous sintered HFs were highly electrically conductive and able to sustain high filtration pressures, making them valuable membranes for micro-filtration applications. Particularly, the SS HF membranes and filters developed in this study presented appropriate pore size distributions for bacteria and yeast rejection toward beverage sterilization applications. In addition, the optimization of the mixed particles’ aspect and size ratio could lead to the development of composite HFs with enhanced mechanical properties and defined pore geometry and porosity for filtration applications, or that act as an electrically conductive diffuser in electro-catalytic reactors.

## Supplementary Materials

The following are available online at http://www.mdpi.com/2077-0375/7/3/40/s1, Figure S1: Thermal treatment and TGA profile of the green SS_10_ HF in air atmosphere with a heating rate of 10°C.min^-1^, Figure S2: SEM images of the green and sintered SS_10_ HFs at temperature ranging from 650 to 1100°C for 60 or 90 min, Figure S3: SEM images of the green and sintered SS_10/20_ HFs at temperature ranging from 650 to 1100°C for 60 or 90 min, Figure S4: SEM images of the green and sintered SS_10/44_ HFs at temperature ranging from 650 to 1100°C for 60 or 90 min, Figure S5: Carbon and oxygen elemental analysis of the SS_10_ HFs (a and d), SS_10/20_ HFs (b and e) and SS_10/44_ HFs (c and f), Figure S6: Pure water fluxes as a function of the feed pressure: (a), (b) and (c): SS_10_ HFs; (d), (e) and (f): SS_10/20_ HFs; g: SS_10/44_ HFs; Table S1: Size distribution of stainless steel particles, Table S2: Metal powder properties: surface area, total BET surface area, average absolute density and relative density determined versus average bulk density of 316L grade stainless steel (8 g.cm^-3^), Table S3: Spinning conditions.
