# Supplementary material for: Preparation of Porous Stainless Steel Hollow-Fibers through Multi-Modal Particle Size Sintering towards Pore Engineering
Source: Membranes (Basel). 2017 Aug 4;7(3):40. doi: 10.3390/membranes7030040 (PMC5618125; doi:10.3390/membranes7030040)
Supplement: Supplementary file 1 [file membranes-07-00040-s001.pdf]

Supporting Information

Preparation of Porous Stainless Steel Hollow-Fibers through Multi-Modal Particle Size Sintering towards Pore Engineering

Francois-Marie Allioux, Oana David, Miren Etxeberria Benavides, Lingxue Kong, David Alfredo Pacheco Tanaka, Ludovic F. Dumée

1. Tables and Figures

Table S1. Spinning conditions.

Sample	Dope Flow Rate (mL·h⁻¹)	Air Gap (cm)	Bore Flow Rate (mL·h⁻¹)	Bore Composition
SS_{10} HF	300	3	100	Water
$SS_{10/20}$ HF	300	1.5	150	Water
$SS_{10/44}$ HF	300	1.5	100	Water

Table S2. Size distribution of stainless steel particles.

Metal Particles	Equivalent Spherical Diameter in DI Water (μm)			Equivalent Spherical Diameter in IP (μm)		
	d0.1	d0.5	d0.9	d0.1	d0.5	d0.9
SS_{10}	3.1 ± 0.1	5.4 ± 0.1	8.6 ± 0.2	3.3 ± 0.1	6.1 ± 0.1	9.5 ± 0.2
SS_{20}	2.5 ± 0.1	9.3 ± 0.1	20.2 ± 0.4	2.8 ± 0.1	10.8 ± 0.1	25.3 ± 0.2
SS_{44}	17.3 ± 0.5	37.3 ± 1	71.9 ± 1.1	18.3 ± 0.1	39.3 ± 0.2	78.3 ± 0.4

Table S3. Metal powder properties: surface area, total BET surface area, average absolute density and relative density determined versus average bulk density of 316L grade stainless steel (8 g·cm⁻³).

Metal Powder	Surface Area (m²·g⁻¹)	Total BET Surface Area (m²)
SS_{10}	0.034	0.087
SS_{20}	0.036	0.096
SS_{44}	0.052	0.139
	Average Absolute Density (g·cm⁻³)	Relative Density (%)
SS_{10}	4.84	39.46
SS_{20}	3.17	60.41
SS_{44}	4.69	41.42
$SS_{10/20}$	2.45	69.38
$SS_{10/44}$	2.72	66.15

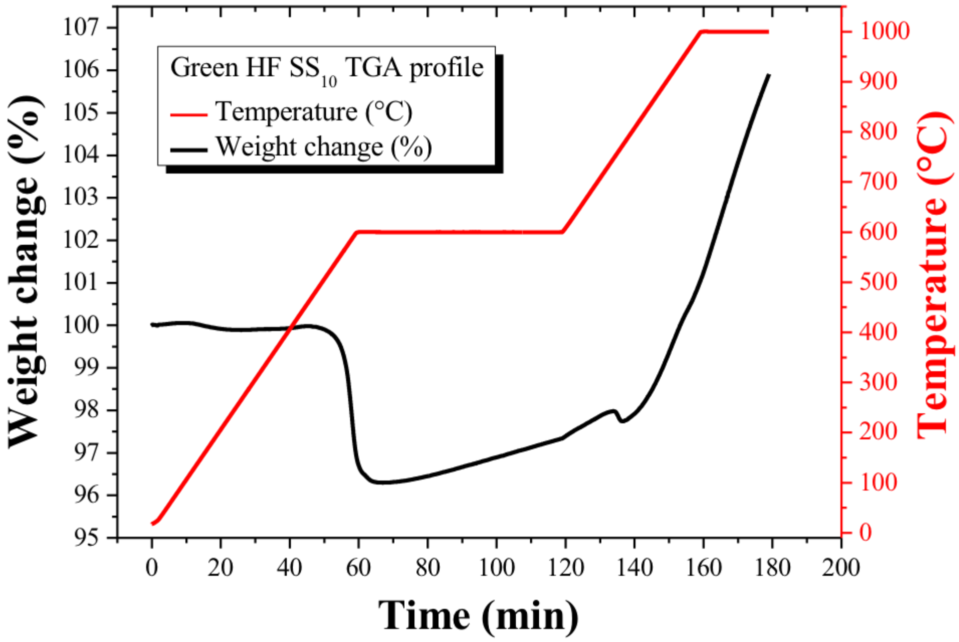

Figure S1. Thermal treatment and TGA profile of the green SS_{10} HF in air atmosphere with a heating rate of 10 °C·min⁻¹.

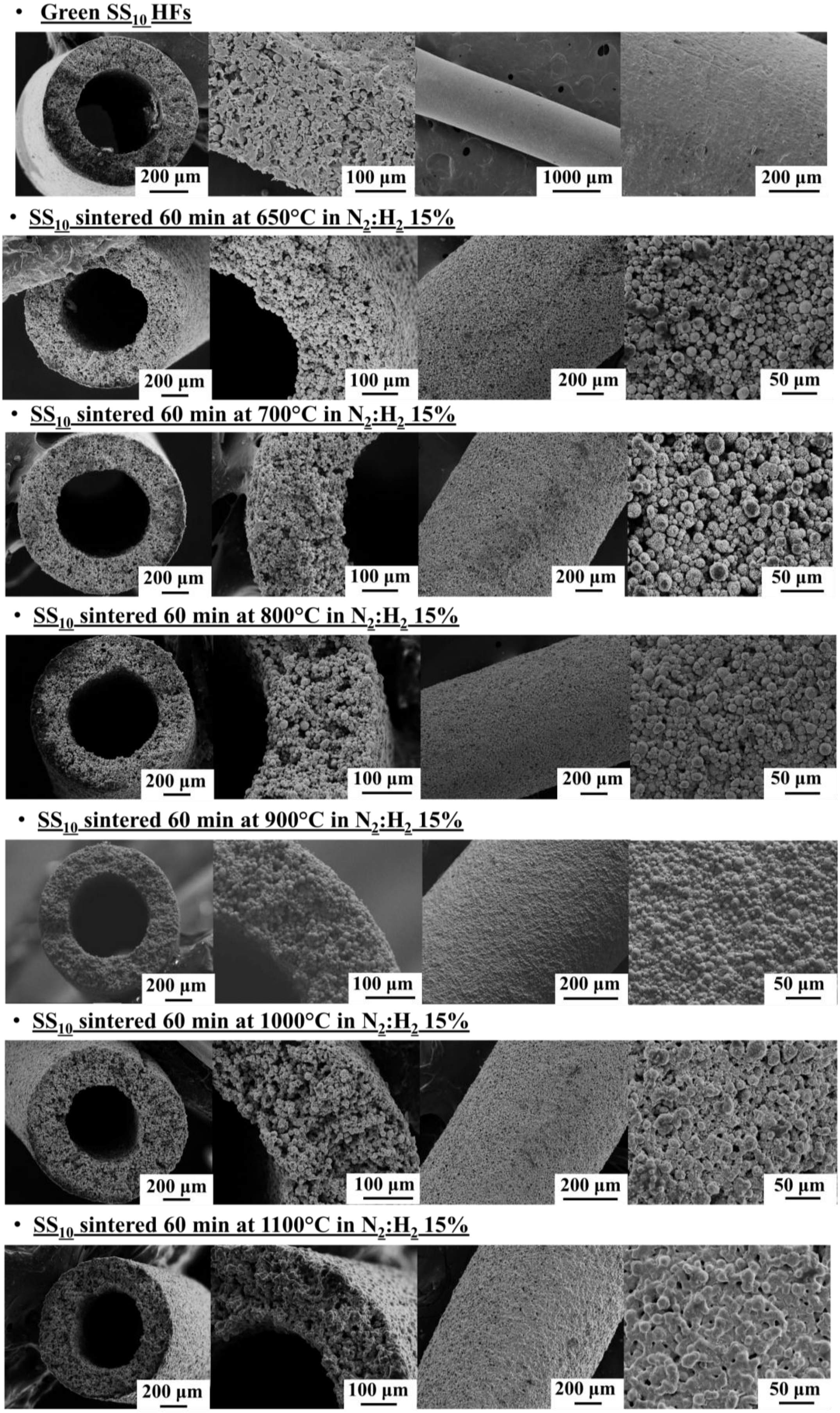

Figure 2. *Cont.*

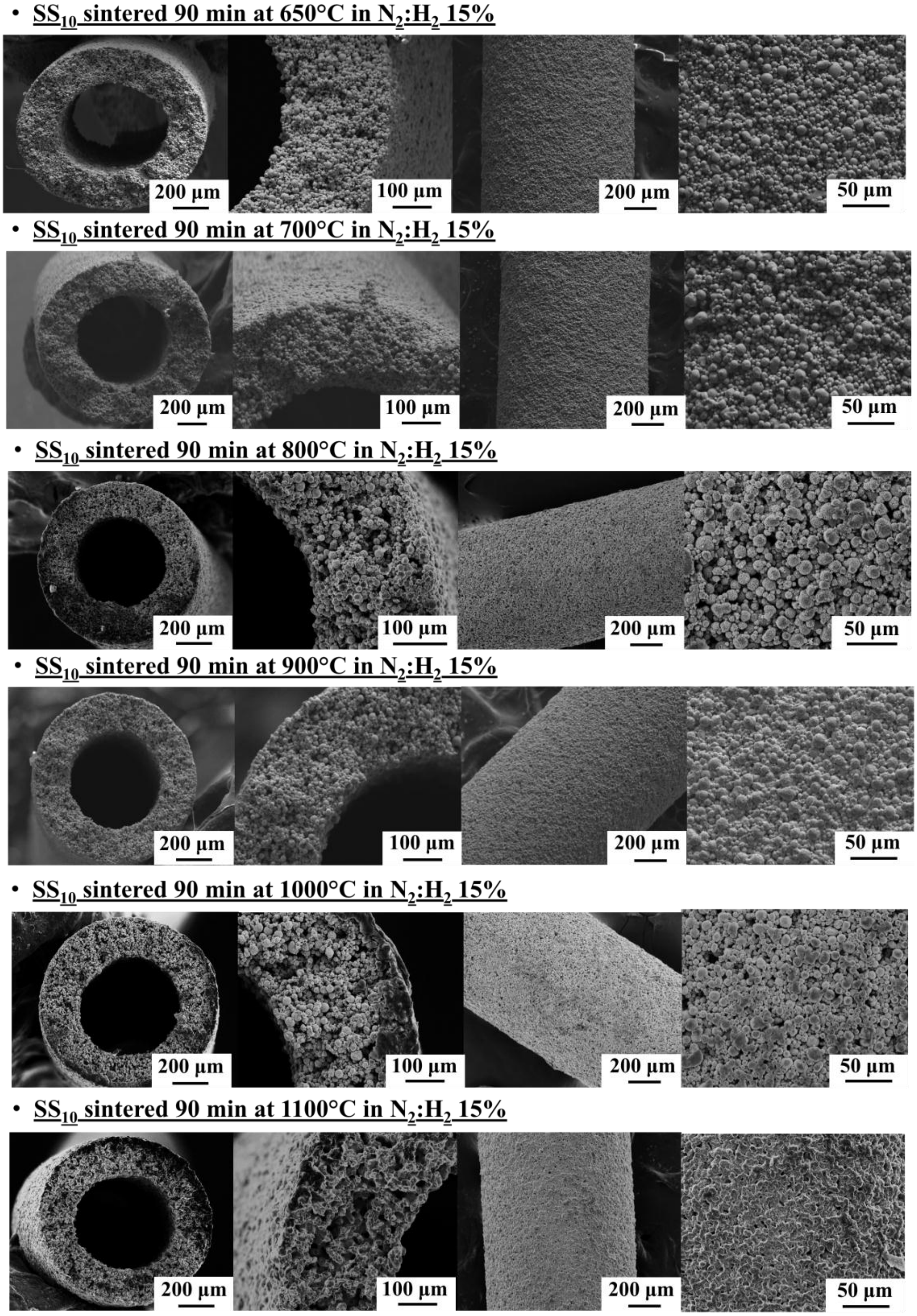

Figure S2. SEM images of the green and sintered SS_{10} HFs at temperature ranging from 650 to 1100 °C for 60 or 90 min.

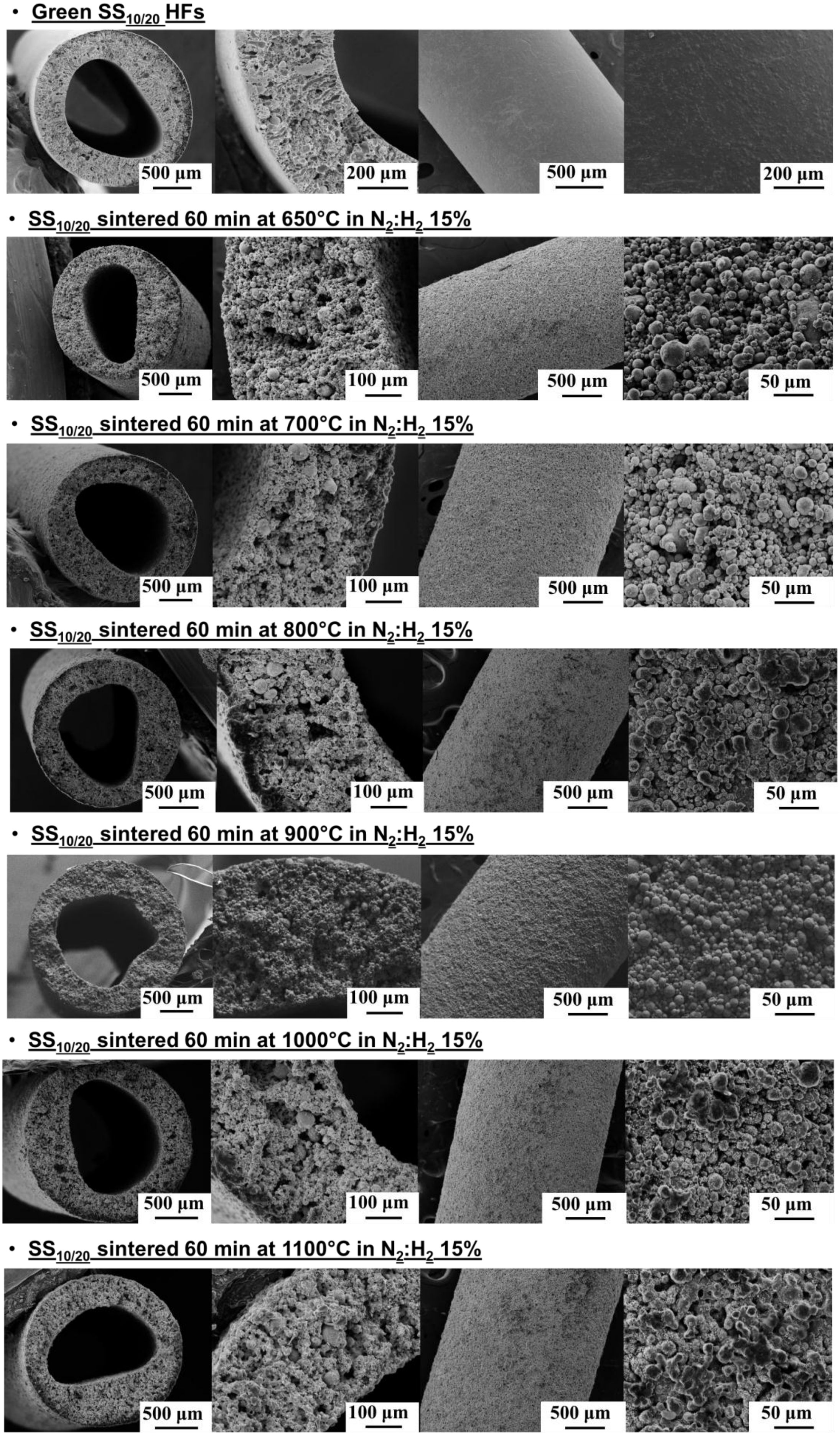

Figure 3. *Cont.*

• **SS$_{10/20}$ sintered 90 min at 650°C in N_2:H_2 15%**

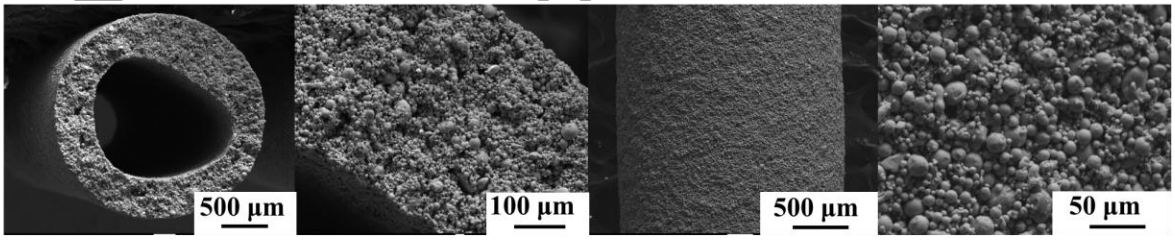

• **SS$_{10/20}$ sintered 90 min at 700°C in N_2:H_2 15%**

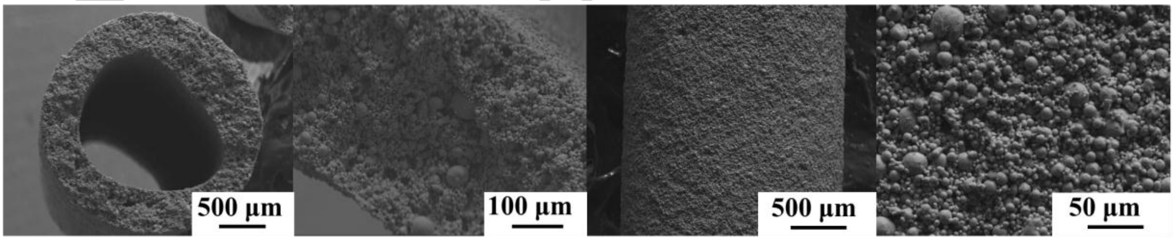

• **SS$_{10/20}$ sintered 90 min at 800°C in N_2:H_2 15%**

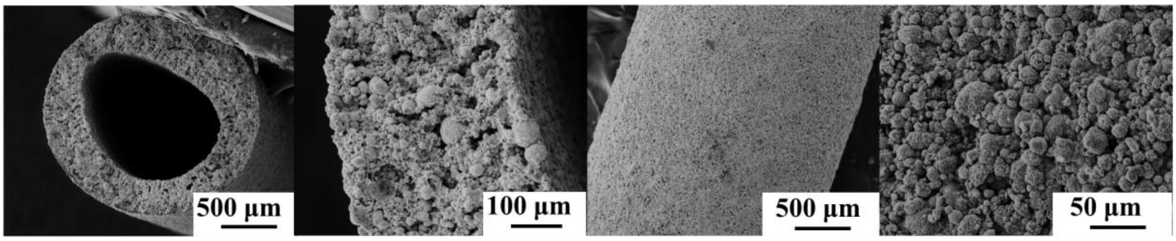

• **SS$_{10/20}$ sintered 90 min at 900°C in N_2:H_2 15%**

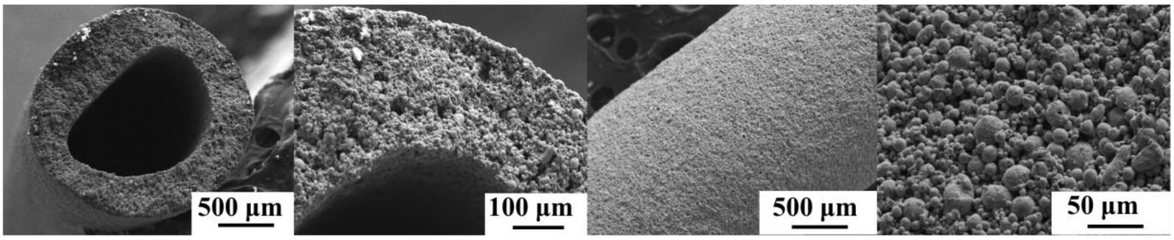

• **SS$_{10/20}$ sintered 90 min at 1000°C in N_2:H_2 15%**

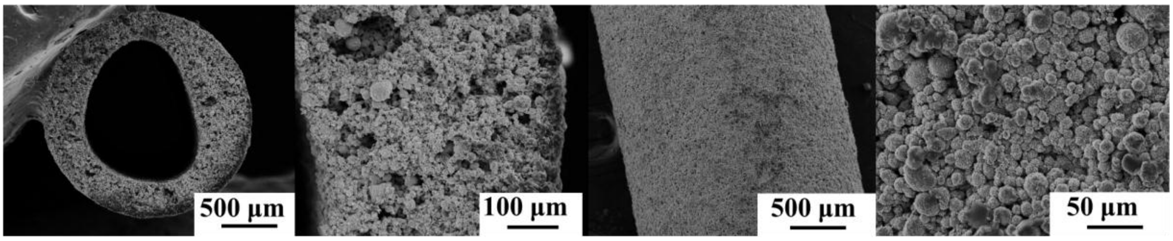

• **SS$_{10/20}$ sintered 90 min at 1100°C in N_2:H_2 15%**

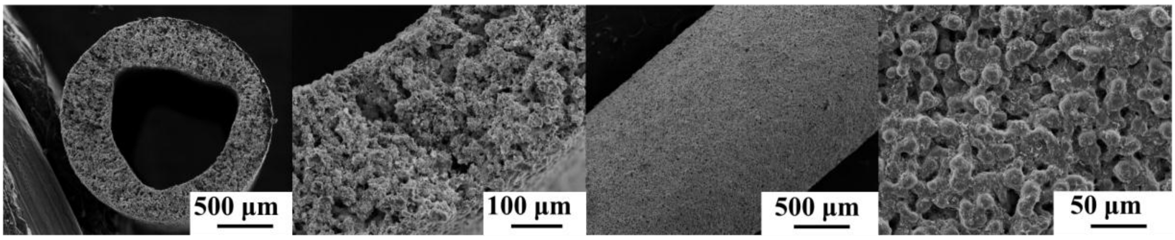

Figure S3. SEM images of the green and sintered SS$_{10/20}$ HFs at temperature ranging from 650 to 1100 °C for 60 or 90 min.

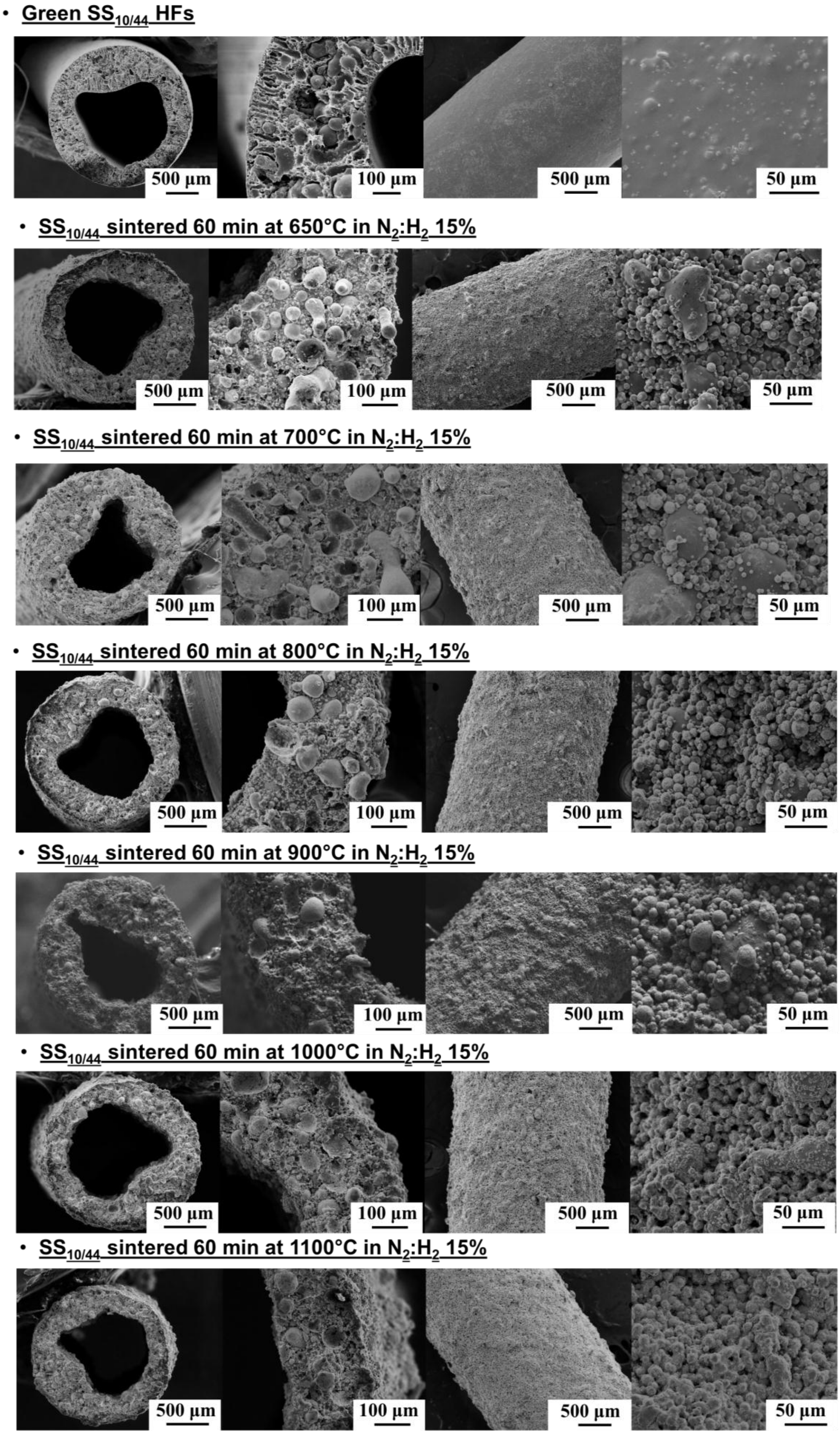

Figure 4. *Cont.*

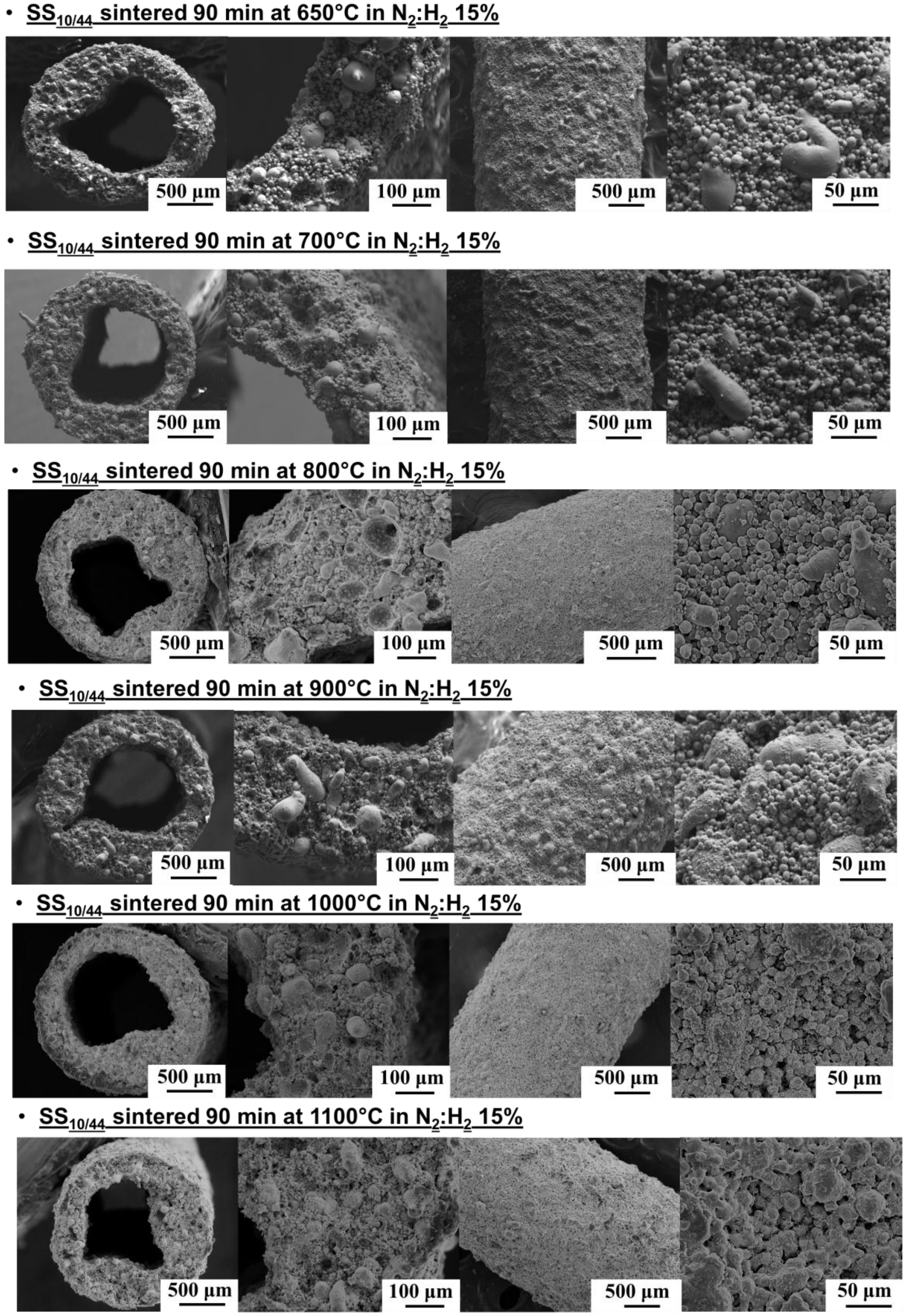

Figure S4. SEM images of the green and sintered $SS_{10/44}$ HFs at temperature ranging from 650 to 1100 °C for 60 or 90 min.

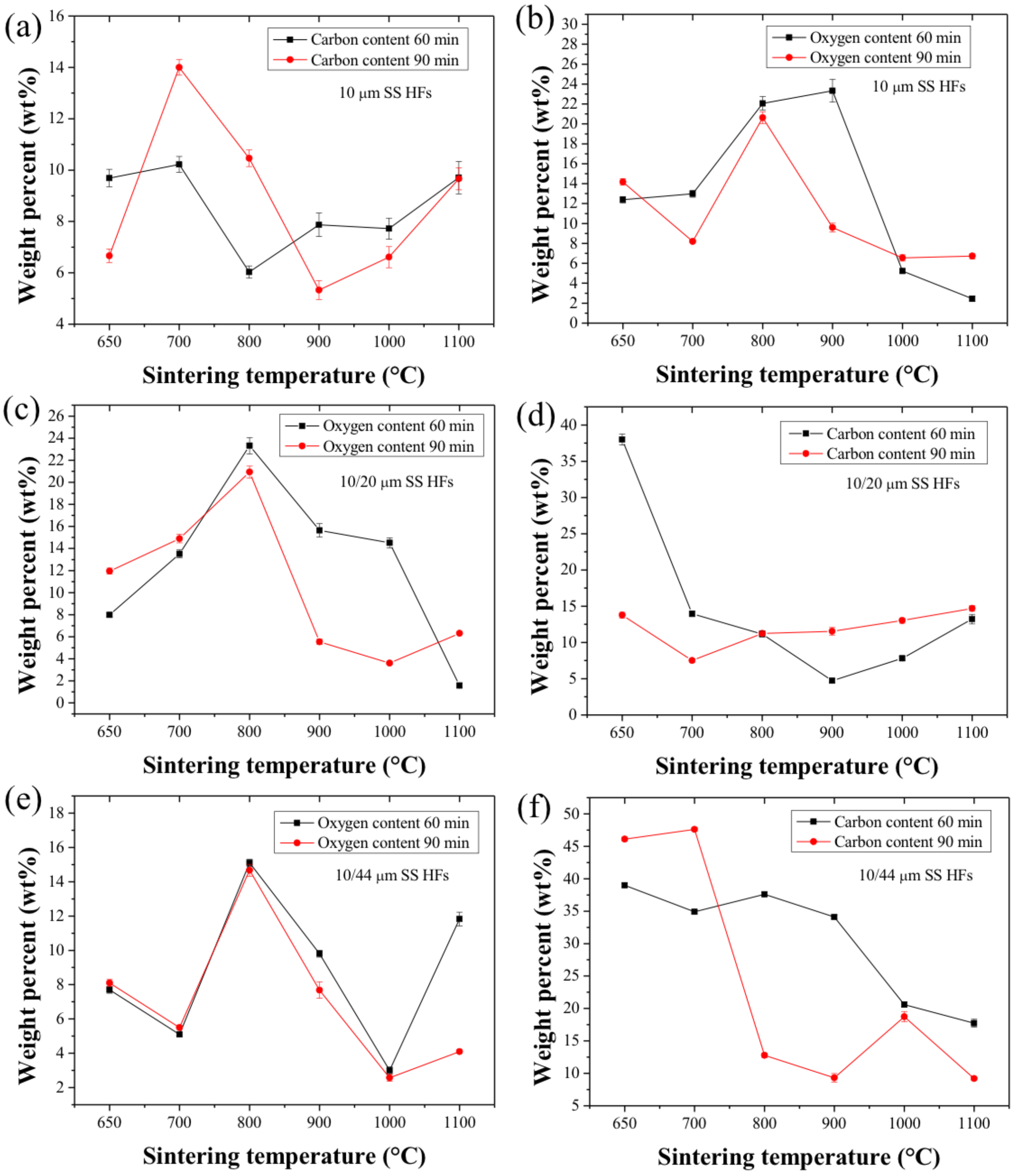

Figure S5. Carbon and oxygen elemental analysis of the SS_{10} HFs (**a**,**d**), $SS_{10/20}$ HFs (**b**,**e**) and $SS_{10/44}$ HFs (**c**,**f**).

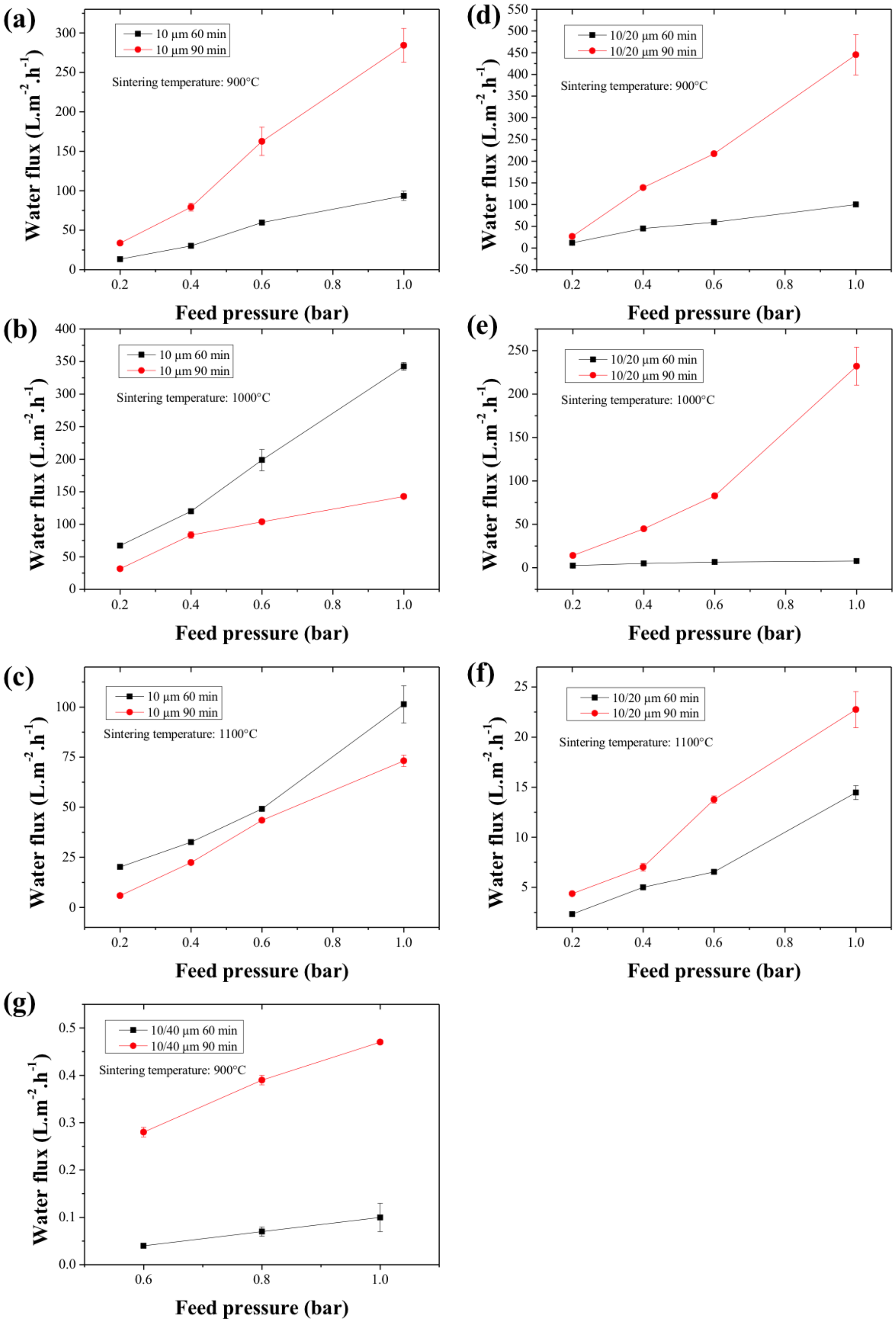

Figure S6. Pure water fluxes as a function of the feed pressure: (**a**), (**b**) and (**c**): SS_{10} HFs; (**d**), (**e**) and (**f**): $SS_{10/20}$ HFs; g: $SS_{10/44}$ HFs.
